# Optical Strain Measurement with Step-Index Polymer Optical Fiber Based on the Phase Measurement of an Intensity-Modulated Signal

**DOI:** 10.3390/s18072319

**Published:** 2018-07-17

**Authors:** Thomas Becker, Olaf Ziemann, Rainer Engelbrecht, Bernhard Schmauss

**Affiliations:** 1 Polymer Optical Fiber Application Center, Technische Hochschule Nürnberg Georg Simon Ohm, Wassertorstraße 10, 90489 Nürnberg, Germany; olaf.ziemann@pofac.th-nuernberg.de (O.Z.); rainer.engelbrecht@pofac.th-nuernberg.de (R.E.); 2 Institute of Microwaves and Photonics (LHFT), Friedrich-Alexander-Universität Erlangen-Nürnberg, Wetterkreuz 15, 91058 Erlangen, Germany; bernhard.schmauss@fau.de

**Keywords:** SI-POF, strain sensing, phase sensing, multi-mode fiber

## Abstract

Polymer optical fibers (POFs) have been proposed for optical strain sensors due to their large elastic strain range compared to glass optical fibers (GOFs). The phase response of a single-mode polymer optical fiber (SM-POF) is well-known in the literature, and depends on the physical deformation of the fiber as well as the impact on the refractive index of the core. In this paper, we investigate the impact of strain on a step-index polymer optical fiber (SI-POF). In particular, we discuss the responsivity of an optical strain sensor which is based on the phase measurement of an intensity-modulated signal. In comparison to the phase response of an SM-POF, we must take additional influences into account. Firstly, the SI-POF is a multi-mode fiber (MMF). Consequently, we not only consider the strain dependence of the refractive index, but also its dependency on the propagation angle $\theta_{z}$. Second, we investigate the phase of an intensity-modulated signal. The development of this modulation phase along the fiber is influenced by modal dispersion, scattering, and attenuation. The modulation phase therefore has no linear dependency on the length of the fiber, even in the unstrained state. For the proper consideration of these effects, we rely on a novel model for step-index multi-mode fibers (SI-MMFs). We expand the model to consider the strain-induced effects, simulate the strain responsivity of the sensor, and compare it to experimental results. This led to the conclusion that the scattering behavior of a SI-POF is strain-dependent, which was further proven by measuring the far field at the end of a SI-POF under different strain conditions.

## 1. Introduction

Glass optical fibers (GOFs) are superior to polymer optical fibers (POFs) for data transmission applications because the lower attenuation and the higher bandwidth allow for higher bit rates and longer transmission distances. Therefore, POF is usually considered only for short-range data transmission [1]. However, for sensing applications, the mentioned limitations of POFs are often not relevant. In fact, there are several advantages which make POFs attractive for sensing tasks. First of all, they are more flexible and robust than GOFs. Due to the large diameter of the core ( 1 mm) of a step-index polymer optical fiber (SI-POF) and the large numerical aperture (NA), the fiber can be excited with an LED, and the fiber tolerates vibrations and smaller displacements or misalignments. Furthermore, an SI-POF and the required components are competitively priced, which offers the possibility of a low-cost sensor design. As a result, several POF-based sensor schemes have been developed for a variety of different applications [2].

A rather simple yet versatile approach is the monitoring of the intensity of the light transmitted by a POF. It has been adopted to measure transversal force [3], liquid levels and the concentration of glycerine solutions [4], as well as vibrations [5]. When the fiber is exposed to constant stress, temperature changes can also be measured with this method [6]. While the evanescent field of a large-core POF is not as strong as that of a single-mode GOF (SM-GOF), its power is sufficient to allow the measurement of moisture [7] and organic gas [8], for example.

POF-based sensors have also been proposed for medical and healthcare applications. The detection of bacteria and other living cells has been demonstrated using POF taper sensors [9]. Another example from this field is the measurement of the particle concentration in water systems based on the light coupling between two POFs to determine the water quality [10].

A technique well known from GOF sensors is the inscription of fiber Bragg gratings (FBGs). FBGs have also successfully been inscribed in POFs, and sensor schemes for the measurement of humidity and temperature [11] as well as strain [12] have been proposed based on this approach. A drawback of the strain sensing using FBGs is the requirement of a complex and expensive measurement setup. However, with structural health monitoring (SHM) gaining more attention, a simple and robust strain measurement technique is desirable. Kiesel et al. investigated the sensitivity of the phase of an electromagnetic wave propagating in a single-mode polymer optical fiber (SM-POF) to strain, and found the initial Young’s modulus to be independent from the strain for strains up to ≈1% [13]. Furthermore, SM-POFs have been proven to endure strain as large as 6% before failure [14] while showing a larger strain responsivity than GOFs [15]. Unfortunately, to the best of our knowledge, there are currently no commercially available SM-POFs.

**Figure 1:**
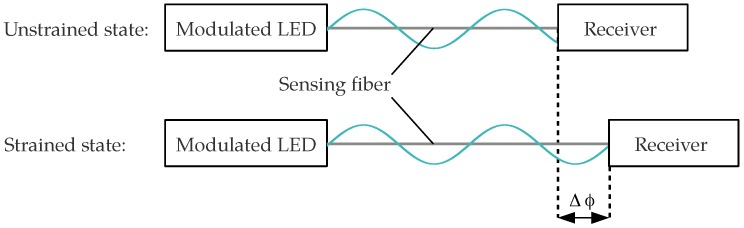
Change of the modulation phase due to strain.

We have proposed an optical strain sensor based on the phase measurement of an intensity-modulated light wave using an SI-POF instead [16,17]. The sensor scheme is simple and competitive. An LED can be used as the light source and an optical receiver is required to convert the intensity-modulated optical power at the end of the fiber into a modulated electrical voltage. When the fiber is strained, the modulation phase at the end of the fiber changes, resulting in a phase shift Δϕ, as depicted in Figure 1. In order to observe the strain state of the sensing fiber, the phase difference between the measured signal and a reference signal can be monitored by a commercially available low-cost integrated circuit (IC). Combined with the general benefits of fiber optical sensors (light weight, immune to electromagnetic interference) and especially the advantages of POF, the sensor design is promising for SHM and other applications. This is especially true since it has been shown that a SI-POF can be strained to more than 40% while maintaining its light-guiding properties [18]. However, in comparison to a SM-POF, the light propagation in a SI-POF is affected by additional effects, including modal dispersion and the angular dependency of the refractive index of the strained fiber. The impact of these effects on the responsivity of the modulation phase to strain has not yet been evaluated. The intention of this paper is to investigate these influences and the consequences for the responsivity of the proposed sensor. 

For an accurate measurement, the sensor requires the strain-induced phase change to be linear to the strain ε:

(1) Δϕ∝ε.

The strain is defined by the ratio of the length change ΔL to the length of the unstrained fiber $L_{0}$:

(2) $$\varepsilon =\frac{\Delta L}{L_{0}}.$$

Therefore, it is clear that the requirement

(3) Δϕ∝ΔL

is given as well. In an ideal fiber, in which only one axial mode propagates, the phase of an intensity-modulated light wave is linear to the length of the fiber:

(4) $$\phi_{ideal}\left(L\right)=2\;\cdot \;\pi \;\cdot \;\frac{L\;\cdot \;n_{0}\;\cdot \;f_{mod}}{c_{0}},$$

where $n_{0}$ is the refractive index of the core, $c_{0}$ is the speed of light in vacuum, and $f_{mod}$ is the modulation frequency. If we consider only the length change due to strain and neglect the impact on the refractive index, the ideal phase change can be expressed as:

(5) $$\Delta \phi_{ideal}=\phi_{ideal}\left(L+\Delta L\right)-\phi_{ideal}\left(L\right)=2\;\cdot \;\pi \;\cdot \;\frac{\Delta L\;\cdot \;n_{0}\;\cdot \;f_{mod}}{c_{0}}=2\;\cdot \;\pi \;\cdot \;\frac{\varepsilon \;\cdot \;L_{0}\;\cdot \;n_{0}\;\cdot \;f_{mod}}{c_{0}}.$$

Hence, the linear dependency of the phase change on the strain is fulfilled. In comparison to the ideal fiber, the development of the modulation phase in an SI-POF is not strictly linear to the length of the fiber. Due to influences such as modal and chromatic dispersion, attenuation, and scattering, this is true even for an unstrained SI-POF. Therefore, we introduce $\phi_{real}\left(L\right)$, which describes the modulation phase in an SI-POF including the mentioned influences:

(6) $$\phi_{real}\left(L\right)=\phi_{ideal}\left(L\right)+\Delta \phi \left(L\right).$$

Δϕ(L) expresses the deviation of the development of the real modulation phase from the ideal phase depending on the length of the fiber. We recently developed a novel fiber model for multi-mode fibers (MMFs) which is yet to be published [19]. It takes all previously mentioned effects into account, which spoil the linear dependency of the modulation phase on the length of the fiber. The model computes the impulse response of the fiber based on scattering and attenuation data obtained from an SI-POF. When transferred to the frequency domain, the phase response allows us to predict the development of the real modulation phase $\phi_{real}\left(L\right)$ along the fiber in the absence of strain. The development of the phase depends on the launching condition and the angular sensitivity of the applied receiver. However, for a realistic launching condition and for a fiber length up to 12 m, the predicted modulation phase $\phi_{real}\left(L\right)$ is only 2% to 3% larger than the ideal phase $\phi_{ideal}\left(L\right)$. Furthermore, the deviation only changes slowly over the length of the fiber. The impact of the development of the real modulation phase in an SI-POF on the phase difference can be expressed as:

(7) $$\Delta \phi_{real}=\phi_{real}\left(L+\Delta L\right)-\phi_{real}\left(L\right).$$

We derived Equation (7) neglecting the impact of strain on the refractive index of the core. Kiesel et al. investigated the consequences of axial strain for the refractive index of the core of an SM-POF [13]. The SM-POF can only guide one mode which is propagating along the axis of the fiber. However, the SI-POF is an MMF and can guide modes up to a maximum propagation angle of $\theta_{c}=\arccos\left(\frac{n_{clad}}{n_{0}}\right)$. If we consider the refractive index of the unstrained core to be $n_{0}=1.49$ and the one of the cladding to be $n_{clad}=1.42$, the maximum guided angle is $\theta_{c}=\arccos\left(\frac{1.42}{1.49}\right)=17.63{}^{\circ }$. Therefore, it is not sufficient to evaluate the impact of axial strain applied to the fiber on the refractive index in the direction of the axis of the fiber. We also investigated the impact on the refractive index in the lateral direction, and can therefore determine the refractive index depending on the propagation angle $\theta_{z}$.

In total, we have to consider the following influences in order to evaluate the full impact of strain on the development of the modulation phase in an SI-POF:

1. Non-linear dependency of the modulation phase on the length of the fiber (Equation (6));
2. Axial and lateral deformation of the fiber;
3. Dependency of the refractive index on the strain ε and on $\theta_{z}$.

The non-linear dependency of the modulation phase on the length of the fiber is already considered by the novel fiber model. The other influences are also integrated into the fiber model to simulate the development of the real modulation phase $\phi_{real}\left(L\right)$ depending on the strain ε. It should be mentioned that the lateral deformation is only considered via the impact on the refractive index. The responsivity of the sensor can then be derived from the relation of the real phase change to the ideal phase change:

(8) $$R=\frac{\Delta \phi_{real}}{\Delta \phi_{ideal}}.$$

$\Delta \phi_{real}$ is the modulation phase change which is obtained from two measurements at different strain states or from the simulations performed with the fiber model.

## 2. Materials and Methods

### 2.1. Physical Deformation of the Fiber

An obvious impact of axial strain on a fiber is the geometrical deformation of the fiber. The length of the fiber in the strained state $L_{s}$ depends on the initial length of the fiber $L_{0}$ and on the axial strain applied to the fiber $\varepsilon_{z}$:

(9) $$L_{s}=\left(1+\varepsilon_{z}\right)\;\cdot \;L_{0}.$$

**Figure 2:**
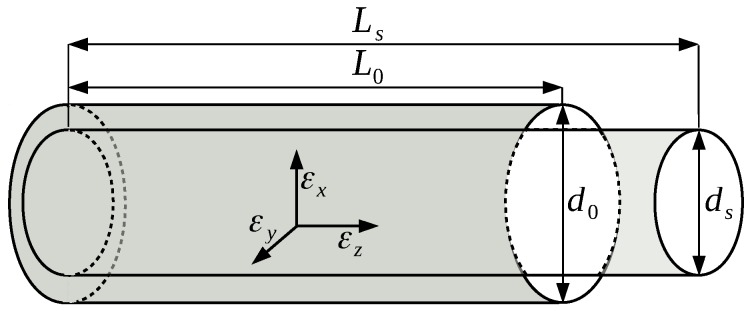
Strain-induced deformation of the fiber.

Furthermore, the applied axial strain leads to a lateral strain in the directions *x* and *y* (Figure 2), which can be obtained with Poisson’s ratio ν:

(10) $$\varepsilon_{x}=\varepsilon_{y}=-\nu \;\cdot \;\varepsilon_{z}.$$

Due to the lateral strain, the fiber faces a lateral deformation as well:

(11) $$d_{s}=\left(1+\varepsilon_{x}\right)\;\cdot \;d_{0}=\left(1+\varepsilon_{y}\right)\;\cdot \;d_{0},$$

where $d_{0}$ is the initial diameter in the unstrained state and $d_{s}$ is the diameter under strain.

The core of an SI-POF is made of polymethylmethacrylate (PMMA). PMMA is a viscoelastic material, which defines its behavior under strain. When a constant stress σ is applied to the fiber, the resulting strain ε is increasing over time. If the fiber is elongated by a constant strain, the resulting stress decreases over time. Finally, Poisson’s ratio in viscoelastic materials also depends on the time [20]. However, in the limited strain range we are about to consider (ε≤1%), we assume a linear dependency of the stress on the strain as stated by Kiesel et al. [13]. Since the Poisson’s ratio of PMMA is also dependent on temperature, the possible values for the Poisson’s ratio for PMMA range from 0.32 to 0.5 for bulk material according to the literature [20].

To the best of our knowledge, the Poisson’s ratio has not been determined for an SI-POF yet and it is likely to differ from bulk material due to the manufacturing process of the fiber. Waxler et al. determined a value of ν=0.345 for a thin layer of PMMA with a thickness of 3.1mm which we will use in the following [21].

### 2.2. Change of the Refractive Index

**Figure 3:**
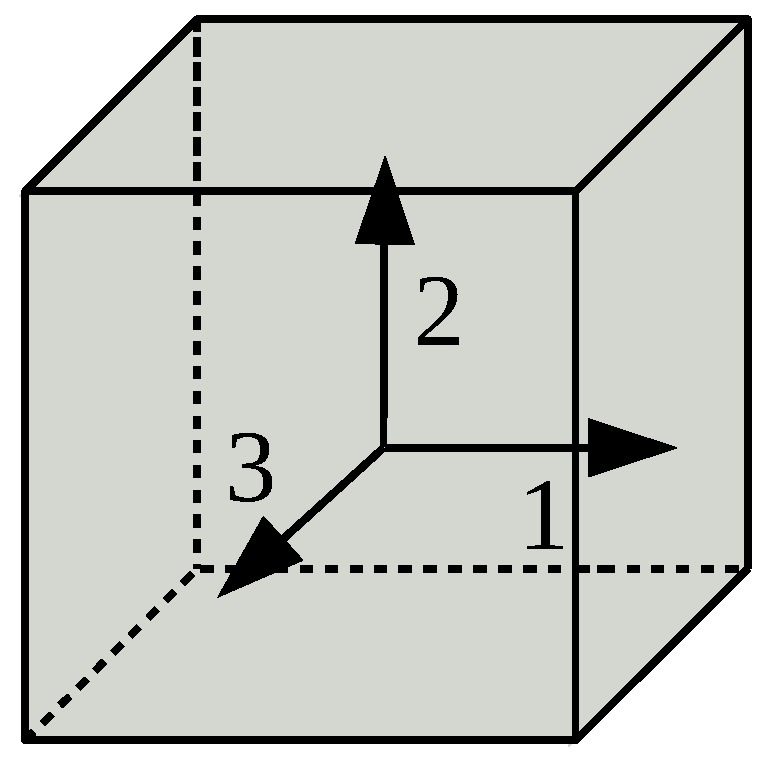
Coordinate system.

For our investigations we consider the core of the fiber to be isotropic in the absence of strain. Hence the refractive index a ray encounters when propagating in an isotropic material is independent of its direction. Since the core of a strained fiber is no longer isotropic, we must consider the direction of a ray and the polarisation of the respective electric field. We consider a ray propagating in the direction 1 (Figure 3). The strain-induced impact on the refractive index of the core for this ray can be calculated from the influence on the electric impermeability tensor B [13,22]. The relation between the refractive index of the core of the unstrained fiber $n_{0}$ and the one of the strained fiber $n_{s}$ depends on the change of the electric impermeability tensor:

(12) $$\frac{1}{n_{s}^{2}}=\frac{1}{n_{0}^{2}}+\frac{\left(\Delta B_{2}+\Delta B_{3}\right)\pm \sqrt{{\left(\Delta B_{2}-\Delta B_{3}\right)}^{2}+4\;\cdot \;\Delta B_{4}^{2}}}{2}.$$

Equation (12) is valid for isotropic materials, and can be used to study the impact of strain on the refractive index by which a ray propagating in direction 1 is affected. Direction 1 can correspond to an arbitrary real direction (*x*, *y*, or *z*), as long as the changes to the components of the electric impermeability tensor can be provided ($\Delta B_{2}$, $\Delta B_{3}$, and $\Delta B_{4}$). The ± operator in front of the square root allows the distinction between different polarizations of the electromagnetic wave that is represented by the considered ray.

Since we assume a linear dependency of the stress on the strain, the change of a specific component of B due to the strain can be expressed with the respective components of the strain-optical tensor p:

(13) $$\Delta B_{m}=\sum_{n=1}^{6}p_{mn}\;\cdot \;\varepsilon_{n}.$$

We use the Voigt notation for the strain components $\varepsilon_{n}$ ($\varepsilon_{1}=\varepsilon_{11}$, $\varepsilon_{2}=\varepsilon_{22}$, $\varepsilon_{3}=\varepsilon_{33}$, $\varepsilon_{4}=\varepsilon_{23}$, $\varepsilon_{5}=\varepsilon_{13}$, and $\varepsilon_{6}=\varepsilon_{12}$). Since we consider the core of the fiber to be isotropic in the unstrained state, p can be expressed by only two coefficients, $p_{11}$ and $p_{12}$ [23]:

(14) $$p=\left(\begin{array}{cccccc} p_{11} & p_{12} & p_{12} & 0 & 0 & 0\\ p_{12} & p_{11} & p_{12} & 0 & 0 & 0\\ p_{12} & p_{12} & p_{11} & 0 & 0 & 0\\ 0 & 0 & 0 & 0.5\;\cdot \;(p_{11}-p_{12}) & 0 & 0\\ 0 & 0 & 0 & 0 & 0.5\;\cdot \;(p_{11}-p_{12}) & 0\\ 0 & 0 & 0 & 0 & 0 & 0.5\;\cdot \;(p_{11}-p_{12}) \end{array}\right).$$

The changes of the relevant components of B are therefore:

(15) $$\begin{array}{c} \Delta B_{2}=p_{11}\;\cdot \;\varepsilon_{2}+p_{12}\;\cdot \;\left(\varepsilon_{1}+\varepsilon_{3}\right),\\ \Delta B_{3}=p_{11}\;\cdot \;\varepsilon_{3}+p_{12}\;\cdot \;\left(\varepsilon_{1}+\varepsilon_{2}\right),\\ \Delta B_{4}=0.5\;\cdot \;\left(p_{11}-p_{12}\right)\;\cdot \;\varepsilon_{4}. \end{array}$$

Furthermore, we can write

(16) $$\Delta B_{2}+\Delta B_{3}=p_{11}\;\cdot \;\left(\varepsilon_{2}+\varepsilon_{3}\right)+p_{12}\;\cdot \;\left(2\;\cdot \;\varepsilon_{1}+\varepsilon_{2}+\varepsilon_{3}\right)$$

and

(17) $$\Delta B_{2}-\Delta B_{3}=p_{11}\;\cdot \;\left(\varepsilon_{2}-\varepsilon_{3}\right)+p_{12}\;\cdot \;\left(\varepsilon_{3}-\varepsilon_{2}\right).$$

We consider a fiber which is strained along the optical axis *z*, and begin with the investigation of the refractive index encountered by a ray propagating along the optical axis. Therefore, we define $\varepsilon_{1}=\varepsilon_{z}$, $\varepsilon_{2}=\varepsilon_{x}=-\nu \;\cdot \;\varepsilon_{z}$, $\varepsilon_{3}=\varepsilon_{y}=-\nu \;\cdot \;\varepsilon_{z}$, and $\varepsilon_{4}=\varepsilon_{5}=\varepsilon_{6}=0$. This leads to

(18) $$\Delta B_{2}+\Delta B_{3}=2\;\cdot \;\varepsilon_{z}\left(p_{12}\;\cdot \;\left(1-\nu \right)-p_{11}\;\cdot \;\nu \right),$$

(19) $$\Delta B_{2}-\Delta B_{3}=0$$

and

(20) $$\Delta B_{4}=0.$$

As we can see, Equation (12) has only one solution in this case since $\Delta B_{2}-\Delta B_{3}=0$. The deformation of the fiber is radially symmetric, and thus the refractive index is independent of the polarization in the considered case. If we insert Equations (18)–(20) into Equation (12), we obtain

(21) $$\frac{1}{n_{s}^{2}}=\frac{1}{n_{0}^{2}}+\varepsilon_{z}\left(p_{12}\;\cdot \;\left(1-\nu \right)-p_{11}\;\cdot \;\nu \right).$$

This expression describes the change of the refractive index encountered by a ray when propagating in the axial direction of the fiber, and is well-known in the literature [24,25].

The next step is the investigation of the impact of strain on the refractive index by which a ray propagating in the lateral directions (*x* and *y*) is affected. Equation (12) is only valid for a ray traveling in the direction 1. Therefore, we reassign the axes. $\varepsilon_{1}=\varepsilon_{y}=-\nu \;\cdot \;\varepsilon_{z}$, $\varepsilon_{2}=\varepsilon_{z}$, and $\varepsilon_{3}=\varepsilon_{x}=-\nu \;\cdot \;\varepsilon_{z}$. With this approach, Equations (16) and (17) lead to

(22) $$\Delta B_{2}+\Delta B_{3}=\varepsilon_{z}\;\cdot \;\left(p_{11}\;\cdot \;\left(1-\nu \right)+p_{12}\;\cdot \;\left(1-3\;\cdot \;\nu \right)\right)$$

and

(23) $$\Delta B_{2}-\Delta B_{3}=\varepsilon_{z}\;\cdot \;\left(1+\nu \right)\;\cdot \;\left(p_{11}-p_{12}\right).$$

Since $\Delta B_{2}-\Delta B_{3}$ is not 0 in the lateral direction, there are two possible solutions to Equation (12), depending on the polarization. As a result of the strain, the core of the fiber shows birefringence. One solution of Equation (12) represents the refractive index for a ray whose electric field oscillates in the xy plane $n_{s,l,xy}$. The other one is for the oscillation in a plane that can be created through the *z* axis $n_{s,l,z*}$.

**Figure 4:**
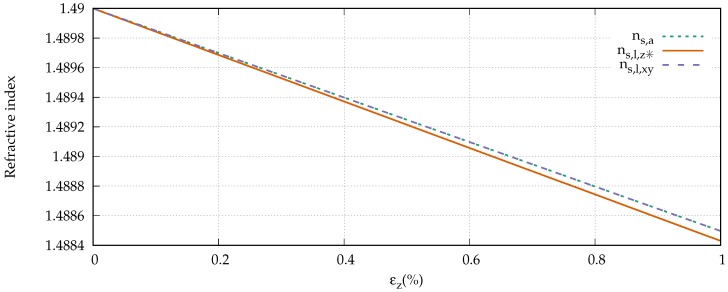
Strain-dependent refractive indices of the core of the fiber.

Waxler et al. experimentally determined the photoelastic constants of PMMA, and found $p_{11}=0.300$, and $p_{12}=0.297$ [26]. With an initial refractive index of the core of $n_{0}=1.49$, we derive the refractive indices for the strained fiber according to Equation (12). Figure 4 shows the development of the refractive indices for the axial direction $n_{s,a}$ and both lateral refractive indices up to a strain of $\varepsilon_{z}=$ 1%. It can be seen that the change is similarly small (≈0.1%) in all three cases. In fact, an axial ray encounters the same refractive index as a lateral ray which is polarized in the xy plane.

Since an SI-POF is known not to maintain the polarization for fibers longer than ≈30 cm [27], it is legitimate to consider a single refractive index for the lateral direction $n_{l}$ which is computed from the mean values of both lateral refractive indices. The fiber model requires the refractive index of the strained fiber for arbitrary angles $\theta_{z}$, which is computed by

(24) $$n_{s}\left(\theta_{z}\right)=\sqrt{{\left(\cos\left(\theta_{z}\right)\;\cdot \;n_{s,a}\right)}^{2}+{\left(\sin\left(\theta_{z}\right)\;\cdot \;n_{s,l}\right)}^{2}}.$$

### 2.3. Measurement Setup and Simulations

**Figure 5:**
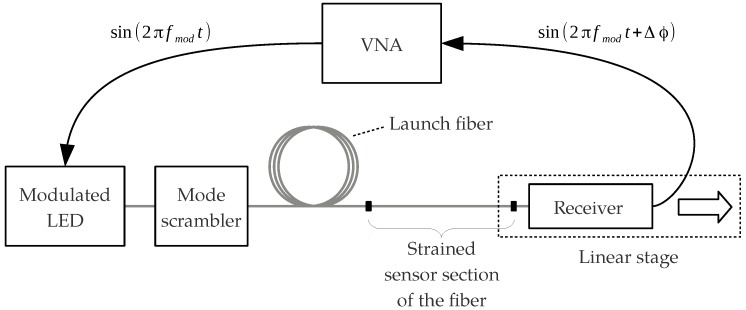
Setup for the strain measurements. VNA: vector network analyzer.

The measurement setup used to investigate the impact of strain on the modulation phase of an intensity-modulated signal is shown in Figure 5. We used a vector network analyzer (VNA) to modulate the power of an LED having a wavelength of 650 nm with a fixed frequency of $f_{mod}=240\;\mathrm{MHz}$. A Y-coupler was used as a mode scrambler to homogenize the slightly irregular far field of the LED, resulting in a nearly Gaussian angular power distribution at both outputs. The investigated fiber was connected to one output, the second output remained unconnected. The fiber was not strained over the full length $L_{Fiber}$. Instead, we only applied strain to the last part of the fiber (sensor section), which had a length of $L_{0}=1\;m$ in the unstrained state.

The preceding unstrained part of the fiber (launch fiber) was altered in length to vary the modal power distribution at the beginning of the sensor section. The aim was to investigate the impact of nonlocal effects on the strain measurement, which are related to the length of the launch fiber. The preceding fiber had the length $L_{p}$ and was coiled with a diameter of $d_{Fiber}=30\;cm$. During the experimental evaluation of the novel fiber model, the development of the modulation phase along a coiled fiber was investigated. The diameter was varied between 10 cm and 40 cm, and was found to have only a negligible impact on the modulation phase [19]. Therefore, we considered the impact of the diameter on the strain measurement to be insignificant as well. In order to investigate the consequences of the length of the launch fiber for the strain measurement, we performed measurements for different values of $L_{p}$. The minimum value of $L_{p}$ cannot be set to 0 m since the fiber has to have a certain length before the first fiber mount in order to attach the required connector. Furthermore, a certain length is necessary to comfortably attach the LED without stressing the fiber. Therefore, the minimum value of $L_{p}$ was chosen to be 1 m. The maximum length of $L_{p}$ was set to 9 m since the modulated signal could still be detected by the receiver without adjusting the power of the LED. The third length of $L_{p}$ was chosen to be 5 m, since it is the average between the minimum and maximum values and allows the recognition of a possible trend in the results.

The sensor section of the fiber was glued into aluminum mounts at the boundaries. While the first mount had a fixed position during the measurement, the second mount at the end of the fiber was attached to a computer-controlled high-precision linear stage with which the axial strain was applied. In order to avoid possible slipping of the fiber in the jacket, bare fibers were used for the measurements. The receiver was placed on the linear stage after the second mount and converted the optical power into an electrical voltage. Apart from a low-impedance amplification included in the receiver, no further signal processing was required and the electrical voltage was directly returned to the VNA. Finally, the VNA was used to determine the phase difference between the transmitted and the received signal. By monitoring this phase difference, we could observe the the impact of strain on the modulation phase.

**Figure 6:**
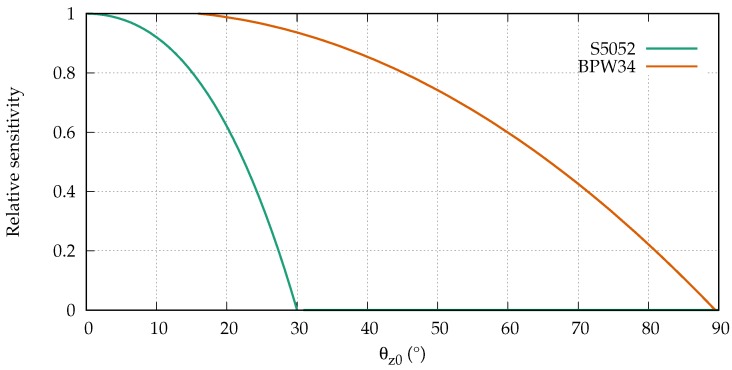
Angular receiver sensitivities.

As mentioned previously, effects such as modal dispersion and scattering affect the development of the modulation phase along an SI-POF. However, the level of influence is affected by the angular acceptance range of the applied receiver. Therefore, we considered two different receivers, both silicon PIN photodiodes, which were used in the simulations and in the measurements. The first was an S5052 [28] from Hamamatsu with a relatively narrow angular sensitivity. The second was a BPW34 [29] from Vishay, which accepts larger angles. The angular sensitivities of both photodiodes taken from the data sheets are depicted in Figure 6, where $\theta_{z0}$ is the propagation angle between a ray and the optical axis outside the fiber.

During a measurement, the strain of the fiber was increased by $\Delta \varepsilon_{z}=$ 0.1% every three seconds, so the maximum strain was $\varepsilon_{z}=$ 1% after 30 s. The measured phase was constant during each stage, thus no creep could be observed. The involved movement of the linear stage for each strain step took less than 100 ms.

The simulations were adjusted to match the measurement setup as closely as possible. The launching condition was matched with the far field at the end of the Y-coupler. We considered Fresnel reflections at the beginning and at the end of the fiber. The scattering and attenuation data used by the model were obtained from a fiber of type Asahi TC-1000, which is the same fiber type used for the measurements (Asahi DB-1000), just with a protective jacket. We simulated the length $L_{p}$ as an unstrained fiber. For the strained last meter of the fiber, we considered the change of the length and the angle-dependent refractive index of the core according to Equations (24) and (9).

## 3. Results and Discussion

### 3.1. Comparison of the Simulated and Measured Responsivities

**Figure 7:**
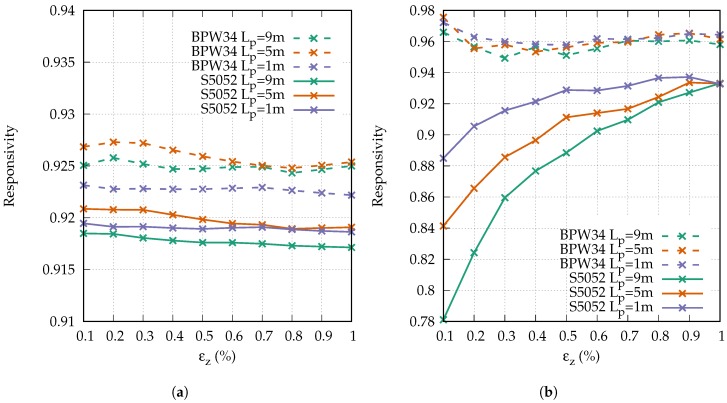
Simulated (**a**) and measured (**b**) responsivities.

Figure 7a shows the responsivity of each strain step simulated with the novel fiber model for both receivers and the three different lengths of the launch fiber preceding the strained range. The responsivity is hereby based on the phase change from step to step, and not on the phase change of the total strain. In all cases, the expected responsivity was in the range of 0.915 to 0.93. This means that the change of the modulation phase for each strain step was smaller than it would be in an ideal fiber.

The dominant influence was the assumed Poisson’s ratio of ν=0.345. The reduction of the diameter of the fiber was not large enough to maintain the volume of the fiber. Hence, the refractive index decreased and therefore the responsivity was reduced in comparison to an ideal fiber. On the other hand, effects such as modal dispersion led to an increased strain responsivity since the modulation phase develops more quickly along an SI-POF than it does along an ideal fiber. The larger the angular acceptance range of the applied receiver, the larger the impact of modal dispersion and the larger the strain responsivity. For that reason, the responsivity was slightly higher for the receiver with the larger angular acceptance range (BPW34) than it was for the receiver with the narrower angular acceptance range (S5052). According to the simulation, the length of the launch fiber and the strain had only a slight influence on the responsivity.

The simulations were repeated, neglecting the angular dependency of the refractive index. The refractive index was set to $n_{s,a}$, independent of the angle $\theta_{z}$. The resulting responsivities barely differed from the depicted values with the angle-dependent refractive index. In fact, the deviation was so small that the responsivities could not be distinguished in the depiction and are therefore not presented.

Since the angular dependency of the refractive index seemed to be negligible, we compared the simulated results to the responsivity of an SM-POF $R_{SM}$. It can be derived by the difference of the optical path lengths that the fundamental mode has to travel in the strained and in the unstrained state, divided by the difference of the optical path lengths neglecting the change of the refractive index:

(25) $$\begin{array}{cc} R_{sm} & =\frac{L_{0}\;\cdot \;\left(1+\varepsilon_{z}\right)\;\cdot \;\left(n_{0}+\Delta n\right)-L_{0}\;\cdot \;n_{0}}{L_{0}\;\cdot \;\varepsilon_{z}\;\cdot \;n_{0}}\\ & =\frac{\left(1+\varepsilon_{z}\right)\;\cdot \;\left(n_{0}+\Delta n\right)-n_{0}}{\varepsilon_{z}\;\cdot \;n_{0}}. \end{array}$$

If we consider the maximum strain of $\varepsilon_{z}=0.01$ and the change of the refractive index as derived for $n_{s,a}$, we obtain a responsivity of the SM-POF of $R_{sm}=0.9$. The simulated values were only ≈1.5% to 3% larger, corresponding to the ratio of how much faster a modulation phase develops along an SI-POF compared to an ideal fiber.

Figure 7b shows the responsivities obtained from the strain measurements. We focus on the results obtained for the receiver with the large angular acceptance range (BPW34) first. All three curves show a value of about 0.96. As in the simulations, the responsivity was only slightly affected by the strain or the length of the launch fiber. Since the measured responsivity was larger than the simulated, one could argue that the Poisson’s ratio used for the simulations might have been too small. It should also be mentioned that the used photoelastic constants were obtained for bulk PMMA. It is therefore possible that the real values for an SI-POF would differ.

The results for the receiver with the smaller angular acceptance range (S5052) differed dramatically from the simulations. As expected, the responsivity was generally smaller than the one for the BPW34. However, it showed a strong dependency on the strain and on the length of the launch fiber. Neither of the dependencies can be explained by any influence that we have considered so far, and are investigated in the next section.

### 3.2. Far Field of the Strained Fiber

**Figure 8:**
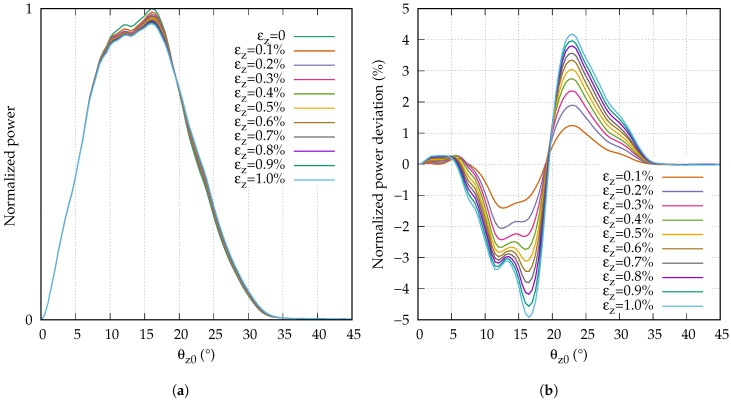
Strain-dependent far field (**a**) and far field changes (**b**).

Seeking a possible explanation for the observed results, we measured the far field at the end of the fiber under different strain conditions. The length of the launch fiber was $L_{p}=9\;m$, and Figure 8a shows the angular power distribution of the far fields normalized to a maximum power of 1. While it can be seen that the peak power decreased with the strain, the changes were too small to be well observable in this representation. Therefore, Figure 8b shows the normalized power deviation of each strain step compared to the far field of the unstrained fiber. The power deviations were normalized to the maximum normalized power level (1). Several conclusions can be drawn from Figure 8b. When the fiber was strained, a part of the power in the range of $\theta_{z0}\approx$ 5${}^{\circ }$ to 18${}^{\circ }$ was shifted towards the range of $\theta_{z0}\approx$ 18${}^{\circ }$ to 35${}^{\circ }$. The stronger the strain, the larger the shifted power. However, the increase of the shifted power per strain step decreased with the strain. 

Unfortunately, we are not able to predict the exact impact of the power shift on the modulation phase since we only observed the far field depending on the strain. It is not fully clear how much power was coupled from one mode to another since by evaluating the far field, we could only observe the sum of all power redistributions. However, we are able to explain some of the consequences for the measurement of the modulation phase.

In order to understand the consequences of the far field changes for the strain measurement, we must first consider the development of the phase of the modulated signal at the end of the fiber. All propagating modes were modulated with the same frequency and the modulation phase of the total signal was determined from the superposition of all modes. The strain-induced scattering of power into higher-order modes affects the modulation phase in two ways. First of all, the mean transit time of the propagating light increases, since some power of lower order modes is shifted to higher order modes. Second, the sensitivities of the used receivers were angle-dependent (Figure 6). The receivers were therefore less sensitive to a certain power in a higher-order mode than they were to the same power in a lower-order mode. Therefore, the conditions on which the phase of the modulated signal depend change during the strain.

Both receivers were affected in the same way by the change of the mean transit time of the propagating light. Since only the strain measurement with the receiver with the narrow angular acceptance range (S5052) showed a dependency on the strain, the change of the mean transit time seemed to have a negligible impact on the modulation phase. Subsequently, the responsivity of the sensor was also unaffected.

Both receivers differed strongly in their angular sensitivity. Consequently, the impact of the strain-induced power shift to higher-order modes had a different impact on the strain measurement, depending on the receiver. The BPW34 has a broad angular acceptance range. The power which was shifted to higher order modes was therefore detected with almost the same sensitivity with which it would have been detected without the power shift. As a result, the responsivity of the sensor was almost independent of the strain. The S5052 has a narrow angular acceptance range. As a result, the power which was shifted to higher-order modes was detected with a much lower sensitivity. In fact, a part of the shifted power was not detected at all. For that reason, the responsivity of the sensor with the S5052 showed a strong strain dependency.

Furthermore, we saw that the amount of power which was shifted per strain step decreased with the strain. As a consequence, the responsivity of the strain measurement with the S5052 changed more quickly at the beginning of the strain range than it did at the end. In fact, the responsivities of all three measurements seemed to converge towards a value of approximately 0.93.

Another interesting detail of the strain measurement with the S5052 was the dependence of the responsivity on the length of the unstrained launch fiber. The longer the fiber, the stronger the change of the responsivity due to the strain. The far field of the launching condition had a narrower range than the far field depicted in Figure 8a, which was recorded after 10 m of fiber. Hence, while propagating, the far field broadened due to the scattering-induced mode coupling. Since the S5052 has a rather narrow angular acceptance range, the impact of strain on the modulation phase was larger for a broader far field.

We did not investigate the origin of the strain-induced power scattering to higher order modes, but at least two causes seem possible. One explanation would be that the polymer chains of the core were aligned due to the strain which affected the scatter process of the fiber. This could also explain why the change decreased with the strain. Another cause could be the introduction of defects at the core–cladding interface, by which the scattering was affected. However, since the whole process is reversible, the latter theory seems unlikely.

## 4. Conclusions

We investigated the impact of strain on an SI-POF and evaluated the consequences for the responsivity of a strain sensor based on the phase measurement of an intensity-modulated signal. We considered the geometrical deformation of the fiber as well as the impact on the angular-dependent refractive index. By integrating the known effects into a novel fiber model, we simulated the responsivity of the sensor for different scenarios and compared the predictions to experimentally obtained data.

The simulations with the model led to the conclusion that the angular-dependency of the refractive index was negligible. Furthermore, the measured responsivities were larger than the simulated ones. This could be caused by the Poisson’s ratio of an SI-POF being actually larger than the assumed value of ν=0.345. It might also be caused by the assumed photoelastic constants. Since they were obtained for bulk PMMA, they could be different for an SI-POF as well.

We showed that the application of axial strain to an SI-POF affected the scattering process encountered by the propagating light. This can have a significant impact on the modulation phase, depending on the length of the fiber and the applied strain. As a consequence, the responsivity of the sensor can significantly change during the strain if a receiver with a narrow angular acceptance range is used. Since this effect depends on the far field at the end of the fiber, its consequences for the strain measurement can hardly be predicted, and are therefore likely to cause a measurement error. The exact change of the scattering process is not known yet. However it was shown that a stable responsivity of the sensor could be achieved by using a receiver with a large angular acceptance range.

## Abbreviations

The following abbreviations are used in this manuscript:

FBG	Fiber Bragg Grating
GOF	Glass Optical Fiber
IC	Integrated Circuit
MMF	Multi-Mode Fiber
NA	Numerical Aperture
PMMA	Polymethylmethacrylate
POF	Polymer Optical Fiber
SI-POF	Step-Index Polymer Optical Fiber
SM-GOF	Single-Mode Glass Optical Fiber
SM-POF	Single-Mode Polymer Optical Fiber
SHM	Structural Health Monitoring
VNA	Vector Network Analyzer
